# Early versus late amniotomy during induction of labor using oxytocin: A randomized controlled trial

**DOI:** 10.1371/journal.pone.0286037

**Published:** 2023-05-25

**Authors:** Ahmed Halouani, Yassine Masmoudi, Rym Hamdaoui, Aymen Hammami, Amel Triki, Anissa Ben Amor

**Affiliations:** 1 Department of Obstetrics and Gynecology, University Hospital Mongi Slim La Marsa, Tunis, Tunisia; 2 Faculty of medicine of Tunis, University Tunis El Manar, Tunis, Tunisia; Poissy-Saint Germain Hospital/Versailles Saint Quentin University, FRANCE

## Abstract

**Objective:**

To assess the effect of early amniotomy on labor duration, maternal and neonatal outcomes during induction of labor (IOL).

**Methods:**

This was a randomized controlled trial, conducted over a period of eight months at a monocentric site. Singleton pregnancies in nulliparous and parous patients with cephalic presentation and Bishop score ≥ 6 were enrolled in the study. One hundred participants were randomized into two groups: early amniotomy (initiating IOL with amniotomy followed by oxytocin) versus late amniotomy (initiating IOL with oxytocin followed by amniotomy 4 hours later). The primary endpoint was the time to active phase (cervical dilation ≥ 5 cm) during IOL. Secondary outcomes were time to vaginal delivery, mode of delivery, and maternal and fetal outcomes.

**Results:**

Early amniotomy reduced time to active phase by 2 hours and 46 minutes compared to the late amniotomy group (3 h 42 min vs. 6 h 28 min; p<0.0001). It also reduced time to vaginal delivery by 2 hours and 52 minutes (5 h 17 min vs. 8 h 9 min; p = 0.0003). The rate of cesarean section (CS) for failed IOL was significantly lower in the early amniotomy group (31.2% vs. 70.0%; p = 0.02), without any significant difference in the overall rate of cesarean section between the two groups (32.0% vs. 40.8%; p = 0.36). There was no significant difference in maternal or fetal outcomes.

**Conclusions:**

Early amniotomy in IOL significantly shortens the time to active phase as well as the overall duration of labor without compromising maternal and neonatal safety.

## Introduction

Induction of labor (IOL) is a common practice in obstetrics [1]. Various indications can lead to IOL. These indications can be either maternal or fetal. The rationale behind inducing labor before spontaneous onset is that the risks associated with the progression of the pregnancy outweigh by far the risks should delivery occur.

In the USA about one in four women undergo IOL. This percentage is expected to rise due to the increasing number of high-risk pregnancies [2].

Oxytocin and amniotomy are two methods used in IOL [3]. In spontaneous onset of labor, there is strong evidence suggesting that amniotomy reduces the overall duration of labor [4]. However, there is lack of evidence to recommend such practice during IOL. Moreover, the optimum timing of rupturing the membranes is still debatable. The effectiveness of amniotomy in IOL has been evaluated in seven randomized controlled trials, which presented heterogeneity in terms of the definition of early and late amniotomy, patient populations, and cervical ripening methods [5–11]. Only one study has focused on amniotomy in patients with a Bishop score ≥ 6, although the Bishop score is a crucial factor in achieving successful IOL [7].

There is ongoing debate about the efficacy of amniotomy in cases of IOL, two meta-analysis have suggested that it has the potential to reduce the duration of labor [12, 13]. Nonetheless, it is important to note that amniotomy also carries an increased risk of complications, including umbilical cord prolapse, chorioamnionitis, and neonatal sepsis [4, 14, 15].

The purpose of this randomized study was to evaluate the impact of early amniotomy on the time to active phase of labor (APL), duration of labor, as well as maternal and neonatal outcomes during IOL.

## Materials and methods

### Ethics statement

The study protocol was approved by the ethical committee of Mongi-Slim University Hospital, La Marsa, Tunisia (approval no. 01/2021). This trial was registered on clinical-trials.org (NCT04731896) on January 2021.

Women admitted for IOL and who met the inclusion criteria were explained the purpose of the study and were invited to participate. Women gave their written consent to take part in the study.

### Study design and participant selection

This is a randomized controlled, non-blind trial. Participants were randomly assigned to two parallel-groups: Group A: Early amniotomy (EA) and Group B: Late amniotomy (LA). The allocation ratio was 1:1.

Women admitted for IOL were considered eligible if they met the following criteria: age ≥ 18 years, a full-term (≥ 37 weeks of gestation), singleton, fetus in cephalic presentation and bishop score of ≥ 6.

Exclusion criteria were women with history of uterine surgery that breached the uterine cavity, previous cesarean section, ruptured membranes, spontaneous onset of labor, macrosomia, severe fetal growth restriction defined as estimated fetal weight by ultrasound < 3rd centile, major fetal abnormalities, maternal HIV, hepatitis B or C, COVID-19 infection or other contraindications to vaginal delivery.

This trial was carried out in the department of obstetrics and gynecology, University Hospital of Mongi Slim La Marsa, Tunis from February 8^th^ to September 30^th^ 2021.

After inclusion, each woman was assigned randomly to either early amniotomy (EA) or late amniotomy (LA) group.

In Group A (EA): women had amniotomy soon after randomization and oxytocin infusion was started 30 minutes later.

In Group B (LA): IOL was initiated with oxytocin infusion, and amniotomy was performed 4 hours later unless deemed necessary earlier (e.g. for non-reassuring fetal heart rate on the cardiotocography (CTG).

Oxytocin was administered intravenously using a syringe infusion pump. The initial dose was 2mUI/min, with a 2mUI increase every 30 minutes. The target was 3–4 uterine contractions per 10 minutes. Once the target was reached, the infusion rate was not increased and kept constant. The maximum infusion rate was 42 mUI/min. The oxytocin infusion was stopped or reduced if hyperstimulation or abnormal CTG occurred. CTG abnormalities were evaluated according to the International Federation of Gynecology and Obstetrics (FIGO) guidelines [16]. Uterine hyperstimulation was defined as five contractions in a 10-minutes interval [17].

The same protocol of oxytocin administration was used in both groups.

Throughout IOL, constant monitoring of fetal heart rate (FHR) and uterine contractions was conducted using an external paper scale CTG and an external tocodynamometer.

Epidural was offered as an option of pain management in the labor ward when the contractions become regular and painful. The monitoring of labor relies on a one-to-one care. To minimize the incidence of chorioamnionitis, cervical examinations were performed every 4 hours in the absence of uterine contractions. However, if the patient experienced the onset of labor, the examinations were conducted hourly, and the midwife documented the findings.

### Study outcomes

The primary outcome was the time spent between initiating the oxytocin infusion and the start of the active phase of labor (APL) defined as cervical dilatation of 5 cm [18–20].

The secondary outcome included: time to vaginal delivery (VD), cesarean delivery rate, intrapartum and postpartum fever, postpartum hemorrhage (PPH), Apgar scores at 1 and 5 min, newborn admission to the neonatal intensive care unit (NICU).

### Sample size and randomization

The sample size was calculated using power calculations to detect a significant reduction in the time to APL. Based on the previous IOL performed in the department, the mean time needed to reach APL was 7± 3 hours and 36 minutes. Using an alpha error of 0.05 and 90% power, aiming to decrease by 150 min the time to APL in EA group, a minimum of 44 patients were needed in each arm [21]. we enrolled 100 women.

A random block allocation sequence was generated with a 1:1 ratio (50 in each group) using a computer-generated randomization program by an independent party, who was not involved in enrolling participants or assigning them to interventions. No stratification was used in this study.

The enrollment was conducted by the resident investigator in this study. The resident investigator’s role was to ensure that the eligibility criteria were met and to provide information about the study and verify the written consent.

### Statistical methods

Quantitative variables with are expressed as mean ± standard deviation (SD), Medians [1st Q- 3rd Q]. Qualitative variables are expressed as percentages. The statistical analysis was carried out using “XLSTAT 2022.3.2.1346”. Data were analyzed using Student-test, Mann-Whitney and Chi square. To compare the two groups, per-protocol analysis was employed. Kaplan-Meier survival analysis with a log-rank test was utilized to compare the primary outcome measure. ANCOVA-test analysis was performed to identify independent factors that may influence the time to APL. All statistical tests were two sided and were performed at a significance level of α = 0.05.

## Results

**Fig 1:**
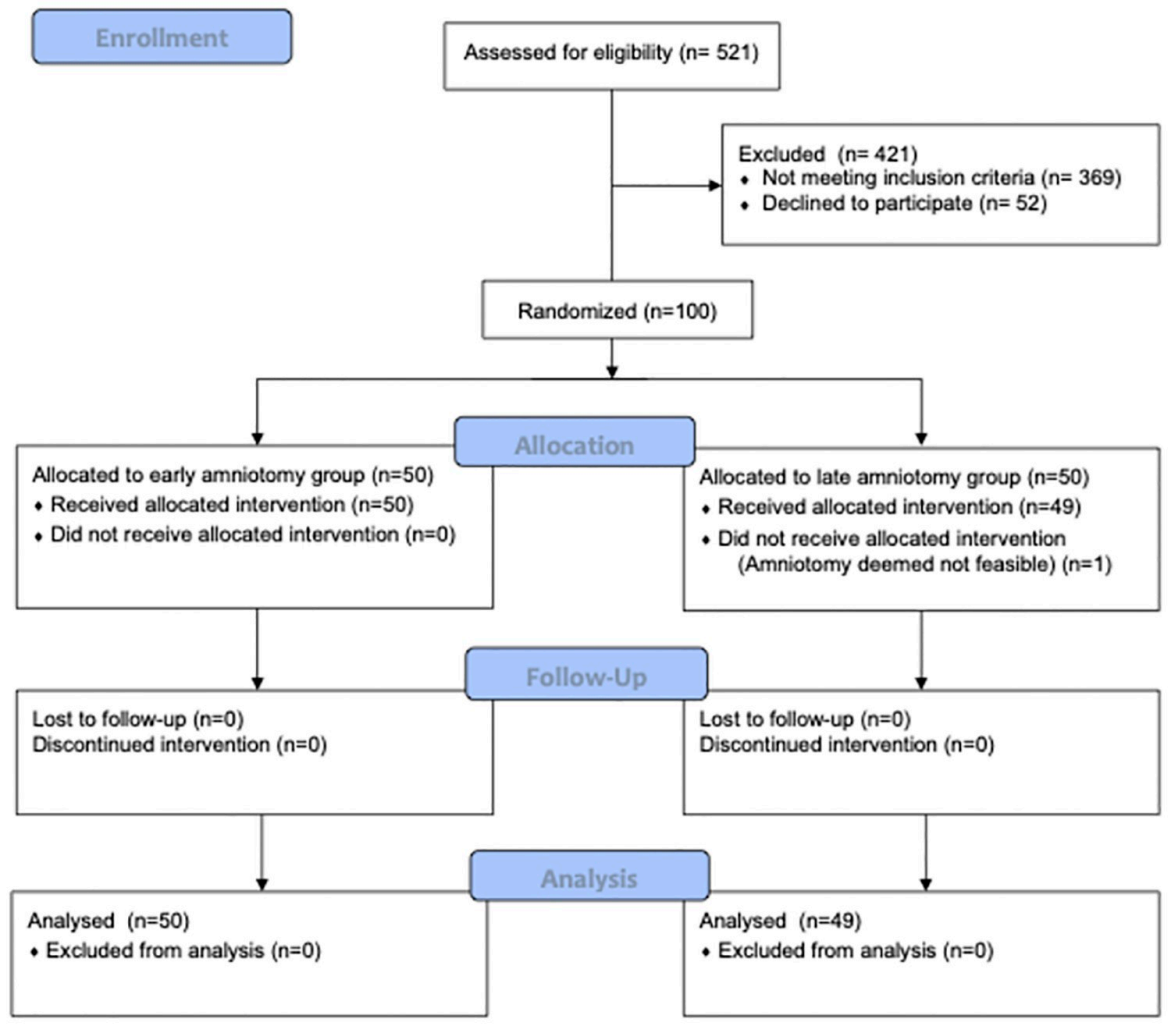
CONSORT 2010 flow diagram.

A total of 521 women was screened, 100 were eligible and agreed to participate in the study. They were randomized equally into two groups: EA or LA. In LA group, in one occasion, the amniotomy was impossible. As we performed a per-protocol analysis, the patient was excluded after randomization. Ninety-nine patients were included in the analysis. The CONSORT flowchart is shown in (Fig 1).

The baseline characteristics, high-risk pregnancy rate, and the mean gestational age are represented in Table 1.

**Table 1:** Baseline characteristics and indications for labor induction in the two groups.

	Early amniotomy (n = 50)	Late amniotomy (n = 49)
Maternal age, y^a^	30 ± 5.6	28 ± 4.7
Body mass index, kg/m^2^^a^	30 ± 4.73	31 ± 4.97
Nulliparous rate, *n* (%)^b^	25 (50.0)	29 (59.1)
Indication for labour induction		
Gestational diabetes, *n* (%)^b^	26 (52.0)	22 (44.9)
Postdated pregnancy, *n* (%)^b^	13 (26.0)	12 (24.5)
Hypertensive disorder of pregnancy, *n* (%)^b^	4 (8.0)	7 (14.3)
Pre-eclampsia, *n* (%)^b^	3 (6.0)	5 (10.2)
Oligoamnios, *n* (%)^b^	4 (8.0)	3 (6.1)
Gestational age, wk^a^	39 ± 7.4	39 ± 8.3
Pre-induction BISHOP’s score^a^	7 ± 0.93	7 ± 0.77
Epidural anesthesia, *n* (%)^b^	38 (76.0)	40 (81.6)

^a^ Data are given as mean ± SD,

^b^ Data are given as number of events (*n*) and percentage

Time to reach APL was shorter in the EA group (3 h 42 min vs. 6 h 28 min; p<0.0001). Additionally, the time to a successful VD was also shorter in the EA group (5 h 17 min vs. 8 h 09 min; p = 0.0003). These findings are observed among both nulliparous and parous patients (Table 2).

**Table 2:** Maternal and neonatal outcome after labor induction in the two groups.

	Early amniotomy (n = 50)	Late amniotomy (n = 49)	p Value
**Time to active phase**, (Mean±SD)	3h 42 min (± 2h 44 min)	6h 28 min (± 2h 40 min)	<0.0001^b^
Median, [1^st^q—3^rd^q]	3h, [2h—6h]	7h, [4h 30min—8h]	
**Nulliparous**	4h 20 min (± 2h 5 min)	7h 11 min (± 2h 28 min)	0.0007^b^
Median, [1^st^q—3^rd^q]	3h 30 min, [2h 45 min—5h 45 min]	7h 30 min, [6h—8h]	
**Parous**	3h 9 min (± 2h 8 min)	5h 39 min (± 2h 44 min)	0.005^b^
Median, [1^st^q—3^rd^q]	2h 30 min, [1h 30 min—3h 30 min]	7h, [3h 56 min—7h 37 min]	
**Time to delivery**, (Mean±SD)	5h 17 min (± 2h 50min)	8h 9 min (± 3h 6 min)	0.0003^b^
Median, [1^st^q—3^rd^q]	4h 45 min, [3h 30 min—6h 22 min]	9h, [5h 35 min—10h]	
**Nulliparous**	6h 34 min (± 3h 6 min)	9h 19 min (± 2h 37 min)	0.001^b^
Median [1^st^q—3^rd^q]	6h [5h—8h]	10h [8h – 10h 45 min]	
**Parous**	4h (± 2h)	7h 5 min (± 3h 13 min)	0.003^b^
Median [1^st^q—3^rd^q]	3h 30 min [3h – 4h 30 min]	8h [5h 30 min– 10h]	
Cesarean delivery, *n*^a^	16	20	0.36^c^
Failed induction of labor, *n* (%)^a^	5 (31.2)	14 (70.0)	0.02^c^
Fetal distress, *n* (%)^a^	4 (25.0)	4 (20.0)	0.15^c^
Arrest of labor, *n* (%)^a^	6 (37.5)	2 (10.0)	0.19^c^
Cord prolapse, *n* (%)^a^	1 (6.3)	0 (0.0)	0.25^c^
Chorioamnionitis, *n* (%)^a^	2 (4.0)	1 (2.0)	0.5^c^
Postpartum hemorrhage, *n* (%)^a^	2 (4.0)	6 (12.2)	0.06^c^
5-minute Apgar score, (Mean±SD)	9.6 ± 0.57	9.7 ± 0.5	0.92^b^
Median [1^st^q—3^rd^q]	10 [9–10]	10 [9–10]	
Birthweight, (Mean±SD)	3432 g ± 428	3299 g ± 371	0.88^b^
Median [1^st^q—3^rd^q]	3450 g [3150 g—3715 g]	3300 g [3100 g—3500 g]	
Neonatal sepsis, *n* (%)^a^	2 (4.0)	1 (2.0)	0.5^c^

^a^ Data are given as number of events and percentage,

^b^ Student Test,

^c^ Chi-square test

After adjusting for BMI, epidural analgesia, parity, and time of amniotomy, the results of the ANCOVA test indicated that EA had the most significant effect on reducing the time to reach active labor phase. Furthermore, the analysis found that nulliparity had a significant impact on lengthening the latent phase of labor (Table 3).

**Table 3:** Independent factors affecting duration of active labor: ANCOVA analysis.

Variables	value	standard error	t	p-value	lower bound (95%)	upper bound (95%)
Amniotomy-EA	-0.483	0.100	-4.822	**<0.0001**	-0.683	-0.283
Amniotomy-LA	0.000	0.000				
Nulliparous	0.272	0.120	2.268	**0.026**	0.033	0.511
Parity = 2	0.050	0.120	0.413	0.681	-0.190	0.289
Parity ≥3	0.000	0.000				
Epidural analgesia-No	0.068	0.100	0.683	0.497	-0.131	0.268
Epidural analgesia-Yes	0.000	0.000				

**Fig 2:**
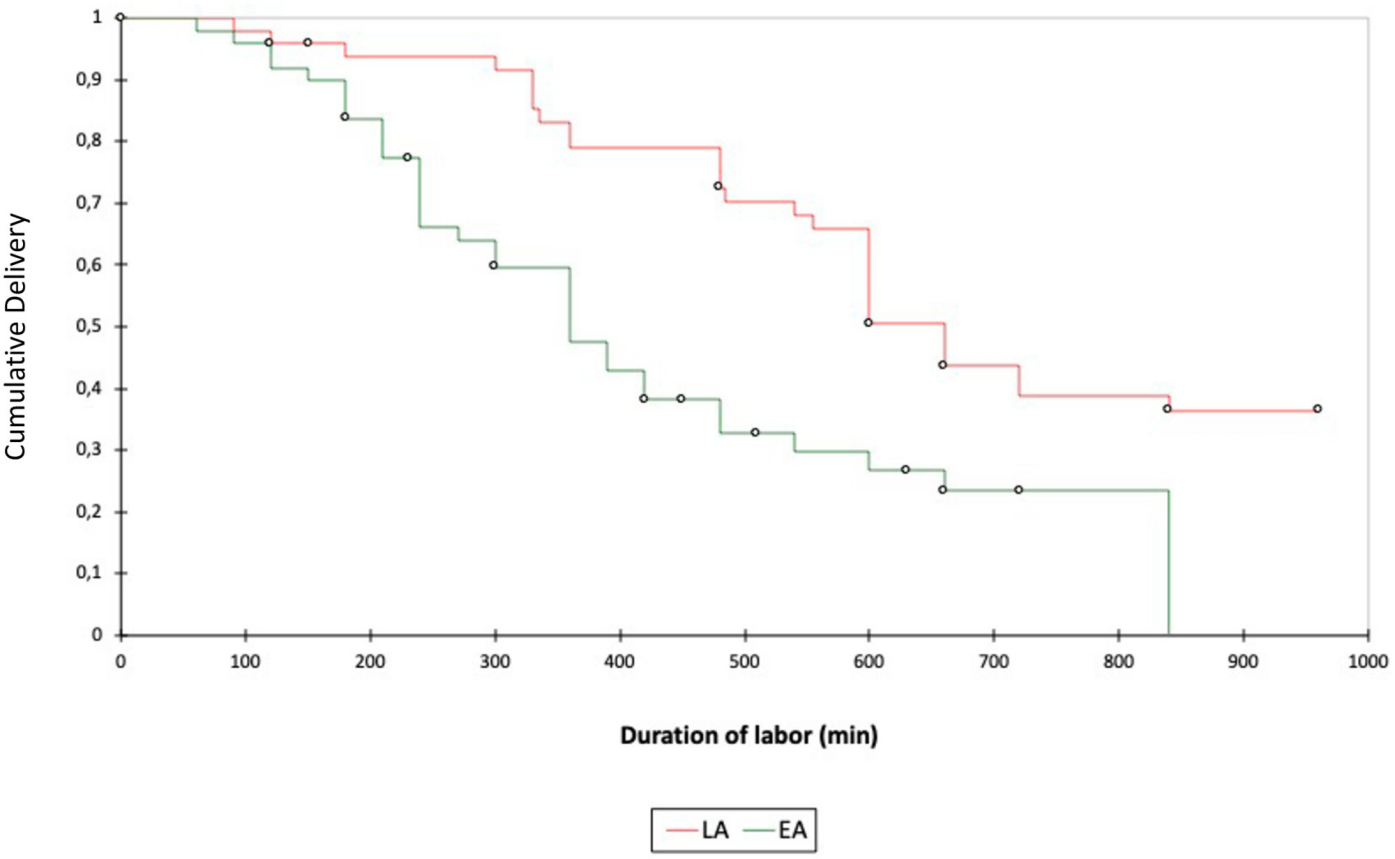
Kaplan Meier analysis results.

The results of the Kaplan-Meier survival analysis with log-rank test revealed that the duration of both the time to APL and the time to VD were significantly shorter in the EA group; p = 0.001 (Fig 2).

There was a significative lower rate of CS for failed IOL in EA compared to LA group (31.2% vs. 70.0%; p = 0.02). However, no significant difference between both groups regarding the overall rate of CS was observed. The main indication of CS in both groups was failed IOL.

Although the difference was not statistically significant, the EA group exhibited a lower incidence of PPH compared to the LA group (4.0% versus 12.2%; p = 0.06). Cord prolapse occurred in only one case in the EA group. The fetal outcomes between the two groups were found to be comparable (Table 2).

## Discussion

The main objective of this study was to evaluate the impact of EA on the time to active phase and the duration of labor, maternal and neonatal outcomes during IOL.

We observed a significant reduction in the time to APL and the overall duration of labor with EA as compared to LA in IOL. Moreover, the rate of caesareans section for failed IOL was significantly lower in the early amniotomy group, without any significant difference in the overall rate of CS between the two groups. These benefits were associated with no significant difference between both groups regarding maternal and neonatal outcomes.

Our study presents several strengths including randomization, the implementation of the CONSORT recommendation, the classification by parity, a well codified oxytocin administration protocol, and a clear description of early and late amniotomy.

However, this study has its limitations. The primary outcome measure of our study was the time to the APL, as successful IOL is defined as achieving this phase after oxytocin and unsuccessful IOL is defined as the inability to reach it, even though the aim of IOL is natural childbirth. Nevertheless, there is no consensus on the definition of the APL among international societies, leading to conflicting interpretations of successful IOL. For instance, the American college of obstetricians and gynecologists (ACOG) defines it as cervical dilation greater than 6 cm [22], while the World Health Organization (WHO) defines it as cervical dilation greater than 5 cm [18]. Moreover, using cervical examination as the primary outcome measure may result in inter-observer variation since cervical dilatation is a subjective measurement. These limitations emphasize the need to standardize the definition of the APL and to identify more objective measures for assessing the success of IOL. Additionally, the study’s monocentric and non-blinded nature is also a limitation that should be considered.

Two meta-analyses, focused on the comparison between LA and EA [12, 13]. Nonetheless, both analyses had a limited number of studies, with one including four trials and the second including seven. Upon closer examination of the studies included in those two meta-analyses, it was apparent that there was a significant issue with the variability of definitions utilized for "early" and "late" amniotomy. Only Makarem et al and Bostanci et al had employed the same definition of LA, which was spontaneous rupture of membranes [5, 10]. However, this definition was subject to significant variation among women. As a result, this variability may have contributed to inconsistent findings and limited the comparability of results. To promote consistency in our study, we adopted the same definitions of early and late amniotomy as the most recent publication that focused on the effect of amniotomy in IOL [7]. The use of standardized definitions and terminology in future studies would have enabled us to draw more relevant conclusions.

The assessment of cervical ripeness using the BISHOP score is a crucial aspect in achieving successful IOL. In this study, our aim was to investigate the impact of amniotomy on IOL success by defining a BISHOP score greater than six. By doing so, we could obtain a more precise evaluation of the actual effect of amniotomy on the success of IOL. The use of a specific BISHOP score in our study provided valuable insights into the effectiveness of amniotomy, which could facilitate better interpretation and comparability of results, leading to more robust conclusions.

In previous clinical trials, a shorter duration of labor was observed in the EA group in comparison to LA group (5–10), except for the studies conducted by Levy et al and Lee et al which did not demonstrate a significant difference in the duration of labor between the two groups [11, 23]. Our study’s findings are in line with previous trials inƒ°dicating that amniotomy can hasten the process of labor during vaginal delivery (Table 4).

**Table 4:** Comparison of studies regarding early or late amniotomy during induction of labor.

	Definition		Labor duration		p-value
	Early amniotomy	Late amniotomy	Early amniotomy	Late amniotomy	
Mercer el al (1995)	Immediate amniotomy or as soon as it was deemed safe and feasible	Deferred until 5 cm dilation	13h 18 min	17h 48 min	0.001
Levy et al (2002)	After expulsion of Foley catheter, immediate amniotomy unless unsuitable	When there were regular contractions or cervical modifications	18h 18 min	7h 24 min	0.22
Macones et al (2012)	Amniotomy at ≤ 4 cm	Amniotomy at > 4 cm	19h	21h 18 min	0.002
G-Gervais et al (2012)	Oxytocin infusion and amniotomy both started within 1 hour after admission	Oxytocin infusion was started but amniotomy was delayed for 4 hours or performed earlier if deemed necessary			
Nulliparous			10h 54 min	15h	0.01
Parous			6h 18 min	7h 42 min	0.04
Makarem et al (2013)	Amniotomy at 3 cm	Spontaneous rupture	9h 43 min	13h 36 min	0.002
Bostanci et al (2018)	Amniotomy at 3 cm	Spontaneous rupture	13h 43 min 7h 33 min*	21h 43 min 16h 08 min*	<0.05 <0.05
Bala et al (2018)	Amniotomy was performed 30–60 min before the oxytocin infusion	Amniotomy was performed 4–8 after the oxytocin infusion	7h 21 min	11h 39 min	<0.0001
Our study:	Amniotomy was performed 30–60 min before the oxytocin infusion	Amniotomy was performed 4–6 after the oxytocin infusion	5h 17 min 3h 42 min*	8h 09 min 6h 28 min*	0.0003 <0.0001
Nulliparous			6h 34 min 4h 20 min*	9h 19 min 7h 11 min*	0.001 0.0007
Parous			4h 3h 09 min*	7h 05 min 5h 39 min*	0.003 0.005

*: Time to active phase of labor

The question arises whether EA has an impact on the CS rate compared to late amniotomy in cases of IOL. In their meta-analysis, Kim et al reported no significant difference in the rate of CS between early and late amniotomy groups (RR, 1.09; 95% CI, 0.80–1.49) [13]. Our results are in agreement with these findings (32.0% vs. 40.8%; p = 0.36). Only Levy et al. and Bala et al. found that early amniotomy was associated with a higher CS rate [7, 11]. In the first trial, the authors suggest that the high rate of CS is due to chorioamnionitis in the EA group. The vaginal examinations performed hourly might have contributed to the increased number of chorioamnionitis, ultimately leading to an increased rate of emergency CS.

Induction of labor (IOL) has been associated with postpartum hemorrhage (PPH), primarily caused by oxytocin infusion and prolonged labor [24, 25]. In our study, 8.1% of the overall IOL population experienced PPH with no significant difference between groups. Oxytocin infusion was closely monitored using a syringe pump and EA was found effective in reducing labor duration and preventing PPH. Moreover, there was no difference between groups in chorioamnionitis and cord prolapse incidence, consistent with literature [5–11, 23].

In the context of labor induction, it is crucial to prioritize maternal and fetal safety over a shorter duration of labor. Our trial confirms the safety and effectiveness of early amniotomy compared to late amniotomy during IOL. Early amniotomy decreases the time to APL and VD, and reduces the cesarean delivery rate due to failed IOL, without increasing the overall cesarean delivery rate or causing any significant difference in maternal or fetal outcomes. The use of this procedure can reduce the mobilization time of medical staff assigned to monitor patients in the delivery room and better organize work in light of the increasing indications for labor induction. However, further studies are needed to evaluate patient satisfaction.

## Conclusion

In women with a favorable cervix, Early amniotomy following induction of labor has been shown to reduce the time to active phase and the total duration of labor, as well as decrease the incidence of cesareans section due to failed Induction of labor. These benefits are observed without any significant difference in maternal or fetal outcomes, when compared to late amniotomy performed 4 hours after initiating induction of labor with oxytocin.

## Supporting information

S1 File Ethical approval. (PDF)

S2 File Study protocol. (DOCX)

S3 File CONSORT 2010 checklist. (DOC)

S4 File Tables and figures. (PDF)
