# Supplementary material for: Early versus late amniotomy during induction of labor using oxytocin: A randomized controlled trial
Source: PLoS One. 2023 May 25;18(5):e0286037. doi: 10.1371/journal.pone.0286037 (PMC10212086; doi:10.1371/journal.pone.0286037)
Supplement: S1 File — (PDF) [file pone.0286037.s001.pdf]

Ministry of Health
University Hospital Mongi Slim La Marsa
Ethical Committee

Certificate

The ethical committee of Mongi Slim hospital gathered on the 8th of January 2021, and examined the request for the following research project:

"Early versus late amniotomy during induction of labor using oxytocin: A randomized trial"

This study was presented by Yassine MASMOUDI MD (Department of Gynecology and Obstetrics).

No ethical breaches were found within this project.

The accord was granted under the number 01/2021, present in this session were:

Pr Lamia BEN JEMAA

CHU Mongi Slim La Marsa
Service des Maladies Congénitales et Héréditaires
Chef de Services
Pr. Lamia BEN JEMAA

Pr Mhammed Sami MEBAZAA

Ministère de la Santé
CHU Mongi Slim La Marsa
Pr M'hamed Sami Mebazaa
Président du Comité Medical

MINISTÈRE DE LA SANTÉ PUBLIQUE
CENTRE HOSPITALO-UNIVERSITAIRE MONGI SLIM LA MARSA
SERVICE DE GYNÉCOLOGIE OBSTÉTRIQUE

Déclenchement du travail : Amniotomie précoce versus amniotomie tardive

ETUDE PROSPECTIVE RANDOMISEE

RESPONSABLE : DR. HALOUANI AHMED
RESIDENT RESPONSABLE : MASMOUDI YASSINE

Université Tunis El Manar, Faculté de médecine de Tunis.

Approved

CHU Mongi Slim La Marsa
Service des Maladies Congénitales et Héréditaires
Chef de Services
Pr. Lamia BEN JEMAA

Ministère de la santé
Hôpital Mongi Slim, La Marsa
Comité d'éthique

Attestation

Le comité d'éthique de l'hôpital Mongi Slim la Marsa s'est réuni le 08 Janvier 2021

et a examiné la demande d'avis du projet de recherche suivant :

"Déclenchement du travail : Amniotomie précoce versus amniotomie tardive : étude prospective randomisée".

Ce travail a été présenté par *Dr.* MASMOUDI Yassine (Service Gynécologie).

Le projet ne soulève pas de problème éthique particulier.

L'accord a été enregistré sous le numéro 01/2021 ; ont participé à cette réunion :

Pr Lamia BEN JEMAA

C H U Mongi Slim La M[illegible]
Service des Maladies Congénitales et Héréditaires
Chef de Service
Pr. Lamia B[illegible]

Pr Mhamed Sami MEBAZAA

Ministère de la Santé
CHU Mongi Slim La Marsa
Pr M'hamed Sami Mebazaa
Président du Comité Medical
