# Supplementary material for: Early versus late amniotomy during induction of labor using oxytocin: A randomized controlled trial
Source: PLoS One. 2023 May 25;18(5):e0286037. doi: 10.1371/journal.pone.0286037 (PMC10212086; doi:10.1371/journal.pone.0286037)
Supplement: S2 File — (DOCX) [file pone.0286037.s002.docx]

**Title: Early versus late amniotomy during induction of labor using oxytocin: A randomized controlled trial.**

**Trial registration:** The trial has been registered in ClinicalTrials.gov.

Trial title: Early versus late amniotomy during labor induction in women with Bishop's Score of ≥ 6.

ClinicalTrials.gov Identifier: NCT04731896

URL : https://clinicaltrials.gov/ct2/show/NCT04731896?cond=NCT04731896&draw=2&rank=1

**Funding:** No funding to declare.

**Roles and responsibilities:**

**Amel Triki, MD^1,2^:** Design, implement and supervising the clinical trial.
Head department of Gynecology and obstetrics, University hospital Mongi Slim la Marsa. Professor Faculty of medicine Tunis.

**Ahmed Halouani, MD^1,2^:** Design, implement and supervising the clinical trial.

**Anissa Ben Amor, MD^1,2^:** Supervising the clinical trial.

**Yassine Masmoudi, Resident^1,2^:** Data Analysis.

**Rym Hamdaoui, Resident^1,2^:** Investigator.

**Lamia Khalfa, Medical secretary^1^:** Data collection.

**Midwives of the unit^1^**: Labor monitoring, data recording.

**Nurses of the labor ward^1^:** Manage the oxytocin infusion.

**Affiliations**:

1. Gynecology and Obstetrics department, University Hospital Mongi Slim La Marsa.

2. Faculty of medicine of Tunis, University Tunis El Manar.

1. **Introduction:**

Background and rationale:

Induction of labour (IOL) is a common practice in obstetrics(1). It involves the artificial initiation of uterine contractions before their spontaneous onset in order to achieve a vaginal delivery. IOL is mainly indicated in during the third trimester when there is a risk to the fetus or the mother to continue the pregnancy.

Amniotomy, also known as artificial rupture of membranes (ARM), and synthetic oxytocin (Syntocinon^®^) remain the main methods used for IOL when the cervix is favorable (Bishop score ≥ 6)(2).

There is ongoing debate about the efficacy of amniotomy in cases of IOL, some authors suggested that it has the potential to reduce the duration of labor(3–8). Nonetheless, it is important to note that amniotomy also carries an increased risk of complications, including umbilical cord prolapse, chorioamnionitis, and neonatal sepsis(9–11).

Objective: To evaluate the impact of early amniotomy on the time to active phase of labor (APL), duration of labor, as well as maternal and neonatal outcomes during IOL.

Trial design: It will be is a randomized controlled, non-blind trial. Participants will be randomly assigned to two parallel-groups: Group A: Early amniotomy (EA) and Group B: Late amniotomy (LA). Random assignment into groups will be computer generated, using 2 equal blocks (ratio 1:1).

**Methods: Participants, interventions, and outcomes**

Study setting:

This trial will be conducted at the obstetrics and gynecology department of the university hospital Mongi Slim la Marsa, Tunis, Tunisia.

Study start date: February 8, 2021.

Participants’ eligibility criteria:

Inclusion criteria:

- Age ≥ 18 years old
- Full term (36 weeks and 3 days of gestation reached)
- Singleton gestation in cephalic presentation
- Bishop score of ≥ 6

Exclusion criteria:

- One or more previous caesarean scars (caesarean section, myomectomy with damage to the uterine cavity)
- Spontaneous rupture of membranes
- Spontaneous onset of labour
- Multiple pregnancies
- Malpresentation (breech, transverse or oblique presentations)
- Polyhydramnios
- Macrosomia (EFW ≥ 4000g)
- Narrow pelvic canal
- Severe foetal growth restriction (EFW ≤3^éme^ Percentile)
- Major foetal abnormalities
- Maternal infections: HIV infection, genital herpes, syphilis, hepatitis B or C
- COVID-19 infection
- Patient aged more than 40 years old
- Severe myopia
- Maternal cardiac disease
- Maternal fever
- Placenta previa
- Meconial amniotic liquid
- Patients declines to participate in the study

Interventions:

Each patient will receive comprehensive information regarding the study objective, and a reflection period will be allocated to facilitate the patient's informed decision-making process before granting their consent.

Prior to randomization, the investigator will conduct a double-check of patients' eligibility, and participants who meet the criteria will be sequentially assigned a study number.

Patients included in the study will be assigned to either early amniotomy (EA) or late amniotomy (LA) group:

In Group A (EA): Amniotomy will be performed soon after randomization, and oxytocin infusion will start 30 minutes later.

In Group B (LA): IOL will be initiated with oxytocin infusion, and amniotomy will be performed 4 hours later unless deemed necessary earlier (e.g. for non-reassuring fetal heart rate on the cardiotocography).

In case of impossibility of membrane rupture, the participant will be excluded.

The oxytocin administration protocol will be implemented utilizing a syringe infusion pump for optimal delivery. The initial dose will be set at rate of 2mUI/min, and it will be increased by and additional 2mUI every 30 minutes. To ensure safety, the infusion rate will not exceed 42 mUI per minute. In case of hyperstimulation or abnormalities in the cardiotocography (CTG) readings, the oxytocin infusion will be halted or reduced accordingly.

The progress of labor will be regularly monitored, with continuous monitoring of the cardiotocography (CTG), cervical dilatation, maternal temperature and blood pressure. All findings and observations will be documented in the patient's medical records.

Once regular and painful contractions have been established, epidural analgesia will be presented as a potential option for pain management.

The monitoring of labor relies on a one-to-one care. Cervical examinations will be performed every 4 hours in the absence of uterine contractions. However, if the patient experienced the onset of labor, the examinations will be conducted hourly, and the midwife documents the findings.

In cases where instrumental deliveries will be required, Simpson type forceps will be utilized. 5 IU of synthetic oxytocin will be administered intravenously at the delivery of the baby's anterior shoulder.

The decision to perform a cesarean section will be made by the supervising team in consultation with the attending consultant and after obtaining the patient's consent.

Concerning the newborn examination, the following parameters will be documented: gender, birth weight, Apgar score at 1, 5, and 10 minutes after birth, as well as signs of post-maturity.

Additionally, any neonatal resuscitation, complications, their reasons, and the duration of admission to the neonatology department will be recorded.

The Apgar score will be used to evaluate the newborn's well-being at birth. A score of seven or less will indicate neonatal distress, while a score of three or less will indicate a state of apparent death, necessitating immediate life-saving measures.

Postpartum examination, complications, and estimated blood loss throughout the entire process will be documented.

Outcomes:

The primary outcome is the duration between initiating the oxytocin infusion and the start of the active phase of labor (APL) defined as cervical dilatation of 5 cm(12,13)(14).

The secondary outcomes are:

- Time to vaginal delivery (VD)
- Caesarean delivery rate
- Intrapartum and postpartum fever (Chorioamnionitis, endometritis…)
- Postpartum haemorrhage (PPH)
- Apgar scores at 1 and 5 min
- Neonatal sepsis and newborn admission to the neonatal intensive care unit (NICU).

Participant timeline:

Sample size:

The sample size was calculated using power calculations to detect a significant reduction in the time to APL. Based on the previous IOL performed in the department, the mean time needed to reach APL was 7± 3 hours and 36 minutes. Using an alpha error of 0.05 and 90 % power, aiming to decrease by 150 min the time to APL in EA group, a minimum of 44 patients are needed in each arm(15). The target is to enroll 100 women.

Recruitment:

The study will be presented to all medical provider to enhance the best adhesion to the protocol. To maximize patient recruitment, women will be sensitized and educated about the indications and potential benefits of the study. Rigorous monitoring of outcomes and maintaining high standards will be prioritized to attract more patients.

**Methods : Assignment of interventions**

Allocation:

A random block allocation sequence will be used to generate with a 1:1 ratio using a computer-generated randomization program by an independent party, who will not be involved in enrolling participants or assigning them to interventions.

Blinding: This study will be a non-blinding study.

**Methods: Data collection, management, and analysis**

We aim to gather all the information’s about the patient base line characteristics, the details of the IOL progress and the maternal and fetal outcomes.

To ensure accuracy and efficiency, we will utilize pre-printed forms to record this information in the medical files (Patient data form). Subsequently, we will enter the recorded data into an Excel spreadsheet, facilitating easy management and analysis.

Quantitative variables with are expressed as mean ± standard deviation (SD), Medians [1st Q- 3rd Q]. Qualitative variables are expressed as percentages. The statistical analysis was carried out using “XLSTAT 2022.3.2.1346”. Data were analyzed using Student-test, Mann-Whitney and Chi square. To compare the two groups, per-protocol analysis was employed. Kaplan-Meier survival analysis with a log-rank test was utilized to compare the primary outcome measure. ANCOVA-test analysis was performed to identify independent factors that may influence the time to APL. All statistical tests were two sided and were performed at a significance level of α=0.05.

**Ethics and dissemination**

The local hospital ethics committee approved the trial protocol: reference number 01/2021.

All personal information about potential and enrolled participants will be collected, anonymized, shared, and maintained in order to protect confidentiality before, during, and after the trial.

References:

1. Tsakiridis I, Mamopoulos A, Athanasiadis A, Dagklis T. Induction of Labor: An Overview of Guidelines. Obstetrical & Gynecological Survey. janv 2020;75(1):61‑72.

2. Bishop EH. PELVIC SCORING FOR ELECTIVE INDUCTION. Obstet Gynecol. août 1964;24:266‑8.

3. Makarem MH, Zahran KM, Abdellah MS, Karen MA. Early amniotomy after vaginal misoprostol for induction of labor: a randomized clinical trial. Arch Gynecol Obstet. août 2013;288(2):261‑5.

4. Mercer BM, McNanley T, O’Brien JM, Randal L, Sibai BM. Early versus late amniotomy for labor induction: A randomized trial. American Journal of Obstetrics and Gynecology. oct 1995;173(4):1321‑5.

5. Bala A, Bagga R, Kalra J, Dutta S. Early versus delayed amniotomy during labor induction with oxytocin in women with Bishop’s score of ≥6: a randomized trial. The Journal of Maternal-Fetal & Neonatal Medicine. 17 nov 2018;31(22):2994‑3001.

6. Macones GA, Cahill A, Stamilio DM, Odibo AO. The efficacy of early amniotomy in nulliparous labor induction: a randomized controlled trial. American Journal of Obstetrics and Gynecology. nov 2012;207(5):403.e1-403.e5.

7. Gagnon-Gervais K, Bujold E, Iglesias MH, Duperron L, Masse A, Mayrand MH, et al. Early versus late amniotomy for labour induction: a randomized controlled trial. The Journal of Maternal-Fetal & Neonatal Medicine. nov 2012;25(11):2326‑9.

8. Bostancı E, Eser A, Yayla Abide C, Kılıccı C, Kucukbas M. Early amniotomy after dinoprostone insert used for the induction of labor: a randomized clinical trial. The Journal of Maternal-Fetal & Neonatal Medicine. 1 févr 2018;31(3):352‑6.

9. Wei S, Wo BL, Qi HP, Xu H, Luo ZC, Roy C, et al. Early amniotomy and early oxytocin for prevention of, or therapy for, delay in first stage spontaneous labour compared with routine care. Cochrane Pregnancy and Childbirth Group, éditeur. Cochrane Database of Systematic Reviews [Internet]. 7 août 2013 [cité 11 mars 2023]; Disponible sur: https://doi.wiley.com/10.1002/14651858.CD006794.pub4

10. Rouse DJ, Weiner SJ, Bloom SL, Varner MW, Spong CY, Ramin SM, et al. Failed Labor Induction: Toward an Objective Diagnosis. Obstetrics & Gynecology. févr 2011;117(2):267‑72.

11. Bump RC, Mattiasson A, Bø K, Brubaker LP, DeLancey JOL, Klarskov P, et al. The standardization of terminology of female pelvic organ prolapse and pelvic floor dysfunction. American Journal of Obstetrics and Gynecology. juill 1996;175(1):10‑7.

12. WHO recommendations: Intrapartum care for a positive childbirth experience [Internet]. Geneva: World Health Organization; 2018 [cité 17 mars 2023]. (WHO Guidelines Approved by the Guidelines Review Committee). Disponible sur: http://www.ncbi.nlm.nih.gov/books/NBK513809/

13. Oladapo O, Diaz V, Bonet M, Abalos E, Thwin S, Souza H, et al. Cervical dilatation patterns of ‘low‐risk’ women with spontaneous labour and normal perinatal outcomes: a systematic review. BJOG: Int J Obstet Gy. juill 2018;125(8):944‑54.

14. Neal JL, Lowe NK, Ahijevych KL, Patrick TE, Cabbage LA, Corwin EJ. “Active Labor” Duration and Dilation Rates Among Low‐Risk, Nulliparous Women With Spontaneous Labor Onset: A Systematic Review. Journal of Midwifery & Women’s Health. 8 juill 2010;55(4):308‑18.

15. Rosner B. Fundamentals of biostatistics. 4th ed. Belmont, Calif: Duxbury Press; 1995. 682 p.
