# Supplementary material for: Early versus late amniotomy during induction of labor using oxytocin: A randomized controlled trial
Source: PLoS One. 2023 May 25;18(5):e0286037. doi: 10.1371/journal.pone.0286037 (PMC10212086; doi:10.1371/journal.pone.0286037)

Table 1: baseline characteristics and indications for labor induction in the two groups.

	Early amniotomy (n=50)	Late amniotomy (n=49)
Maternal age, y[a]	30 ± 5.6	28 ± 4.7
Body mass index, kg/m^{2a}	30 ± 4.73	31 ± 4.97
Nulliparous rate, *n* (%)[b]	25 (50.0)	29 (59.1)
Indication for labour induction		
Gestational diabetes, *n* (%)[b]	26 (52.0)	22 (44.9)
Postdated pregnancy, *n* (%)[b]	13 (26.0)	12 (24.5)
Hypertensive disorder of pregnancy, *n* (%)[b]	4 (8.0)	7 (14.3)
Pre-eclampsia, *n* (%)[b]	3 (6.0)	5 (10.2)
Oligoamnios, *n* (%)[b]	4 (8.0)	3 (6.1)
Gestational age, wk[a]	39 ± 7.4	39 ± 8.3
Pre-induction BISHOP's score[a]	7 ± 0.93	7 ± 0.77
Epidural anesthesia, *n* (%)[b]	38 (76.0)	40 (81.6)

Table 2: Maternal and neonatal outcome after labor induction in the two groups

	Early amniotomy (n=50)	Late amniotomy (n=49)	p Value
**Time to active phase,** (Mean±SD)	3h 42 min (± 2h 44 min)	6h 28 min (± 2h 40 min)	<0.0001[b]
Median, [1st q - 3rd q]	3h, [2h - 6h]	7h, [4h 30min - 8h]	
**Nulliparous**	4h 20 min (± 2h 5 min)	7h 11 min (± 2h 28 min)	0.0007[b]
Median, [1st q - 3rd q]	3h 30 min, [2h 45 min - 5h 45 min]	7h 30 min, [6h - 8h]	
**Parous**	3h 9 min (± 2h 8 min)	5h 39 min (± 2h 44 min)	0.005[b]
Median, [1st q - 3rd q]	2h 30 min, [1h 30 min - 3h 30 min]	7h, [3h 56 min - 7h 37 min]	
**Time to delivery,** (Mean±SD)	5h 17 min (± 2h 50min)	8h 9 min (± 3h 6 min)	0.0003[b]
Median, [1st q - 3rd q]	4h 45 min, [3h 30 min - 6h 22 min]	9h, [5h 35 min - 10h]	
**Nulliparous**	6h 34 min (± 3h 6 min)	9h 19 min (± 2h 37 min)	0.001[b]
Median [1st q - 3rd q]	6h [5h - 8h]	10h [8h – 10h 45 min]	
**Parous**	4h (± 2h)	7h 5 min (± 3h 13 min)	0.003[b]
Median [1st q - 3rd q]	3h 30 min [3h – 4h 30 min]	8h [5h 30 min – 10h]	
Cesarean delivery, *n*[a]	16	20	0.36[c]
Failed induction of labor, *n* (%)[a]	5 (31.2)	14 (70.0)	0.02[c]
Fetal distress, *n* (%)[a]	4 (25.0)	4 (20.0)	0.15[c]
Arrest of labor, *n* (%)[a]	6 (37.5)	2 (10.0)	0.19[c]
Cord prolapse, *n* (%)[a]	1 (6.3)	0 (0.0)	0.25[c]
Chorioamnionitis, *n* (%)[a]	2 (4.0)	1 (2.0)	0.5[c]

Postpartum hemorrhage, *n* (%)[a]	2 (4.0)	6 (12.2)	0.06[c]
5-minute Apgar score, (Mean±SD)	9.6 ± 0.57	9.7 ± 0.5	0.92[b]
Median [1[st]q - 3[rd]q]	10 [9 - 10]	10 [9 - 10]	
Birthweight, (Mean±SD)	3432 g ± 428	3299 g ± 371	0.88[b]
Median [1[st]q - 3[rd]q]	3450 g [3150 g - 3715 g]	3300 g [3100 g - 3500 g]	
Neonatal sepsis, *n* (%)[a]	2 (4.0)	1 (2.0)	0.5[c]

Table 3: Independent factors affecting duration of active labor: ANCOVA analysis

Variables	value	standard error	t	p-value	lower bound (95%)	upper bound (95%)
Amniotomy-EA	-0.483	0.100	-4.822	**<0.0001**	-0.683	-0.283
Amniotomy-LA	0.000	0.000				
Nulliparous	0.272	0.120	2.268	**0.026**	0.033	0.511
Parity = 2	0.050	0.120	0.413	0.681	-0.190	0.289
Parity ≥3	0.000	0.000				
Epidural analgesia-No	0.068	0.100	0.683	0.497	-0.131	0.268
Epidural analgesia-Yes	0.000	0.000				

Table 4: Comparison of studies regarding early or late amniotomy during induction of labor.

		Definition		Labor duration		p-value
		Early amniotomy	Late amniotomy	Early amniotomy	Late amniotomy	
Mercer el al (1995)		Immediate amniotomy or as soon as it was deemed safe and feasible	Deferred until 5 cm dilation	13h 18 min	17h 48 min	0.001
Levy et al (2002)		After expulsion of Foley catheter, immediate amniotomy unless unsuitable	When there were regular contractions or cervical modifications	18h 18 min	7h 24 min	0.22
Macones et al (2012)		Amniotomy at ≤ 4 cm	Amniotomy at > 4 cm	19h	21h 18 min	0.002
G-Gervais et al (2012)		Oxytocin infusion and amniotomy both started within 1 hour after admission	Oxytocin infusion was started but amniotomy was delayed for 4 hours or performed earlier if deemed necessary			
	Nulliparous			10h 54 min	15h	0.01
	Parous			6h 18 min	7h 42 min	0.04
Makarem et al (2013)		Amniotomy at 3 cm	Spontaneous rupture	9h 43 min	13h 36 min	0.002
Bostanci et al (2017)		Amniotomy at 3 cm	Spontaneous rupture	13h 43 min<br>7h 33 min*	21h 43 min<br>16h 08 min*	<0.05<br><0.05
Bala et al (2017)		Amniotomy was performed 30-60 min before the oxytocin infusion	Amniotomy was performed 4-8 after the oxytocin infusion	7h 21 min	11h 39 min	<0.0001
Our study :				5h 17 min<br>3h 42 min*	8h 09 min<br>6h 28 min*	0.0003<br><0.0001

	Nulliparous	Amniotomy was performed 30-60 min before the oxytocin infusion	Amniotomy was performed 4-6 after the oxytocin infusion	6h 34 min<br>4h 20 min*	9h 19 min<br>7h 11 min*	0.001<br>0.0007
	Parous			4h<br>3h 09 min*	7h 05 min<br>5h 39 min*	0.003<br>0.005

Fig1. CONSORT 2010 flow diagram

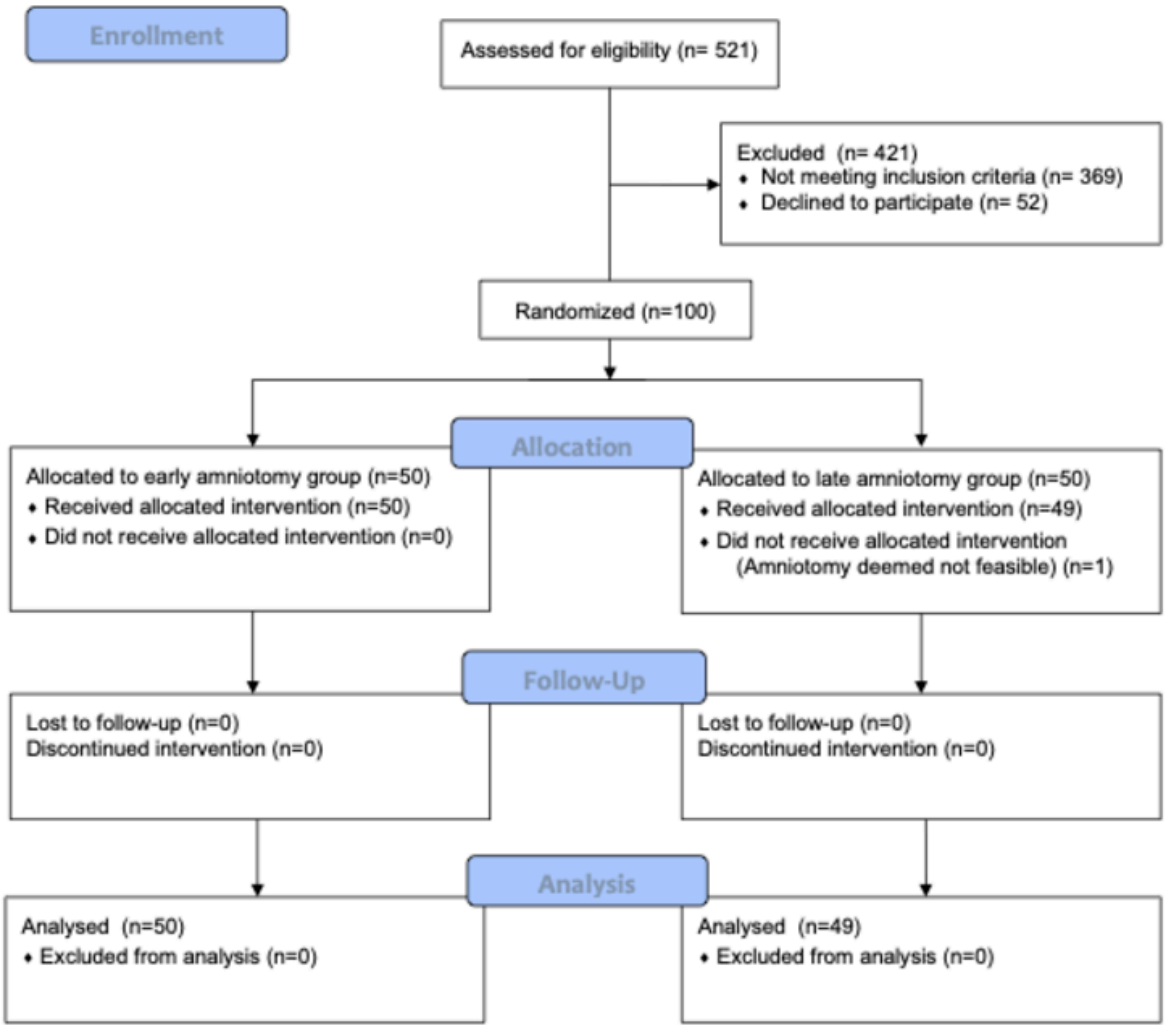

Fig2. Kaplan Meier analysis results.

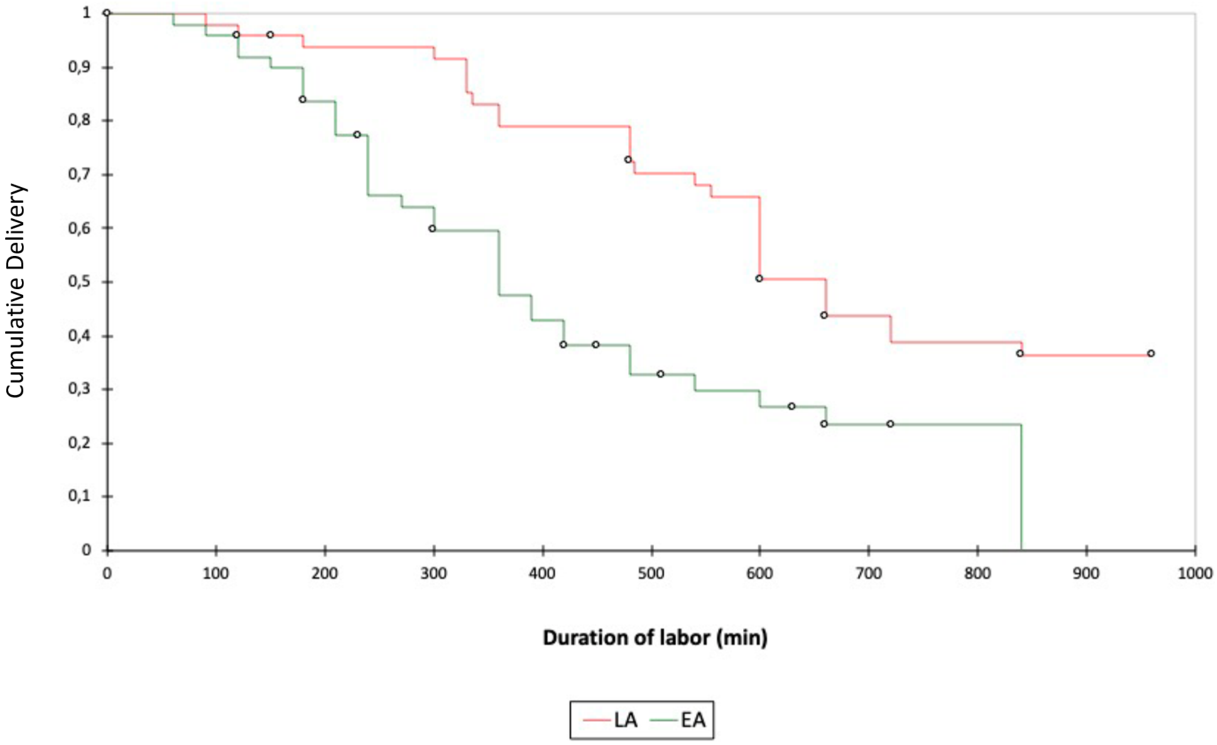

Supplement: S4 File — (PDF) [file pone.0286037.s004.pdf]
